# Empirical study on the construction of a cognitive model of factorization in eighth-grade students

**DOI:** 10.3389/fpsyg.2023.1171352

**Published:** 2023-07-07

**Authors:** Xu Zhangtao, Cai Jiabao, Li Jiale, Li Na, Li Bo

**Affiliations:** School of Mathematics and Statistics, Central China Normal University, Wuhan, China

**Keywords:** factorization, cognitive diagnosis, cognitive models, regression analysis, middle school education

## Abstract

The construction of cognitive models is the basis for cognitive diagnosis, and the cognitive models will change based on the purpose of the study. According to the purpose of mathematical education, the cognitive factorization model is constructed based on the competence and knowledge dimensions. The factorization cognitive model was preliminarily constructed using expert-defined and literature surveys, and a small-scale test was subsequently carried out. The rationality of the cognitive model was tested through verbal reports and the regression of the item's difficulty through the cognitive attributes. The study included a sample of 72 students from two eighth-grade classes in a junior high school located in Wuhan. A diagnosis was made based on the mastery of factorization knowledge and the level of mathematical operation ability of the eighth graders in the cognitive model. Research 1 demonstrates that the construction of the cognitive factorization model is reasonable. Research 2 shows that approximately 79% of students' mathematics operation ability can reach the level of knowledge understanding, 71% of students can reach the level of knowledge transfer, and only 28% of students can reach the level of knowledge innovation.

## Introduction

Frederiksen et al. (1993) classified the evolution of the psychological test theory into two stages: standard test theory and test theory for a new generation of tests. Standard test theory includes classical test theory (CTT), generalizability theory (GT), and item response theory (IRT) (Dai, 2010), which emphasizes measuring and assessing an individual's macro-ability level. However, the psychological significance of depicting the macro-ability level's “statistical structure” is fuzzy. Cognitive diagnostic (CD) is based on project response theory and is more sensitive to the machining process of individual micro psychology, revealing the psychological significance of the “statistical structure” of the macroscopic ability level. The new generation of test theory emphasizes the need to simultaneously assess an individual's macro-level competency and diagnose their micro-psychological processes (Tu et al., 2012). The realization of this process is based on the cognitive model of problem-solving and the psychological measurement model of testing. The cores of cognitive diagnostic assessment theory (CDAT) are the cognitive model and the cognitive diagnosis models (CDMs) (Tu et al., 2019). Currently, there is no uniform standard for the definition of cognitive models worldwide (Petkov and Luiza, 2013). While the development and research of CDMs is a hot topic, both at home and abroad, there are over 60 CDMs that can be used by researchers in contemporary times. The cognitive diagnosis model, which is based on the theory of cognitive diagnosis and combined with the mathematical model, can set the cognitive characteristics specified in the study as the independent variables in the mathematical model and the subjects' responses as the dependent variables in the model to compute the mathematical relationship between the independent variables and the dependent variables and diagnose the subjects' cognitive structure (Deng, 2018). The development of a cognitive model is the foundation of cognitive diagnosis, and its rationality has a direct impact on the accuracy rate. Direct cognitive diagnosis without a cognitive model is akin to entering a large city without a map and stumbling around on crisscrossing roads (Ding et al., 2012). While the number of studies on the construction of cognitive models is relatively small, there is no suitable cognitive model for achievement testing, mainly because the cognitive model is relatively complex, and its construction differs according to different research purposes. It is not only closely related to subject knowledge but also related to the researchers' comprehension of the subject knowledge's internal structure. The order of the development of mathematics knowledge itself is different from the order arranged in the textbook, and there are individual differences in the structure of mathematics knowledge on the minds of students (Yu P., 2018). Owing to these differences, the construction of the cognitive models turned out to be more meaningful. The development of ability is based on the mastery of knowledge, and mastering knowledge itself is also an ability (Yu J., 2018, pp. 80–85). Factorization is one of the most important identical transformations in middle school mathematics, and the factorization process includes many mathematical thinking methods, such as equation thought, whole thought, and so on, which can greatly cultivate students' observation, operation, and creative abilities. This view coincides with the theoretical community's view on the relationship between knowledge and ability. Yu J. (2018) divided the results of knowledge learning into knowledge understanding, knowledge transfer, and knowledge innovation, which represent the three levels of key competence in mathematics disciplines (Yu J., 2018, pp. 80–85). Based on the relationship between knowledge and ability, taking junior high school algebra's ‘factorization' as an example and combining “mathematics key ability level,” which was put forward by Yu J. (2018) eighth graders' factorization cognitive model was constructed together on the knowledge dimension and the ability dimension. The purpose of mathematics teaching is not only to learn existing knowledge; one of its most important purposes is to transfer the acquired knowledge to a new situation, which requires students to learn to solve problems creatively (Shao et al., 1997, pp. 88–89). Mathematical learning relies on understanding; understanding promotes migration, and knowledge innovation is the result of understanding's arrival. There have been many types of research on factorization teaching and studies of the application of cognitive diagnosis. Factorization is one of the most important transformations in middle school mathematics. The process of factorization includes many mathematical methods (Yin, 2016, pp. 40–434), such as equations and whole ideas, which can cultivate students' observation ability, operation ability, creativity, etc. According to existing research, students often make mistakes on the test because they do not understand the meaning of factorization. Presently, the applied research in cognitive diagnosis can be divided into two categories: competence research and subject knowledge research (Dai et al., 2006, pp. 10–11), and the goal of cognitive diagnosis is to help with teaching and personalization of learning (Zhang and Wang, 2016). Therefore, the existing cognitive models have the following characteristics: First, the cognitive model is constructed only from the knowledge dimension and does not reflect the ability dimension in the knowledge dimension. Second, the cognitive model is constructed only from the capability dimension and does not reflect the knowledge dimension in the capability dimension. Finally, the cognitive model constructed by the knowledge dimension fails to reflect the connection between knowledge at a deeper level.

## Cognitive attributes and cognitive models

Cognition and attributes are the basic concepts in the theory of cognitive diagnostic assessment. Cognition refers to the process by which people acquire or apply knowledge. It is the most fundamental aspect of the psychological processes of human beings, including psychological phenomena such as feeling, perception, memory, thinking, imagination, and language (Luo, 2019). Leighton et al. (1999) believed that “attribute” describes the declarative or procedural knowledge required to complete a problem in a certain field. Attributes are divided into physiological attributes (such as gender and height) and psychological attributes (such as feeling, perception, and ability). This theory refers to the psychological attributes of human beings, which represent the implicit psychological traits that affect people's explicit behavior. In this study, attributes specifically refer to the psychological traits of knowledge, skills, and other abilities. The construction of cognitive models requires consideration of cognitive attributes. According to the purpose of mathematical education, the cognitive factorization model is constructed based on the capability and knowledge dimensions.

### Capability dimension attribute analysis

Knowledge understanding, knowledge transfer, and knowledge innovation are the three different patterns into which Professor Yu separated knowledge acquisition outcomes. He claimed that “knowledge is the origin of critical skill development.” Each pattern corresponds to a level of key competence in the discipline, stratification can be said to increase the three modes or stages of core literacy development (Yu J., 2018, pp. 80–85). Knowledge understanding (Yu, 2017) refers to the understanding and mastery of the essence of knowledge, the capacity to comprehend fundamental concepts and principles, and the capacity to carry out simple applications, which is the first level of the key competence of the discipline, which is recorded as B1. Knowledge transfer refers to the capacity to transfer the fundamental knowledge and fundamental skills to various learning circumstances and to use a variety of knowledge and methods to solve problems. It is the discipline's second level of key competence, which is denoted as B2. Knowledge innovation refers to the ability to analyze and judge things mathematically and know the world with mathematical thinking and vision. This is not only the ultimate goal of mathematical education but also the third level of the key competence of the discipline, which is denoted as B3. This article mainly studies the mathematics operation ability of the eighth graders, so the students' operation ability can be divided into three levels: B1, B2, and B3.

### Knowledge dimension attribute analysis

Factorization is one of the most important constant transformations in middle school mathematics. It is clearly stipulated in the “Compulsory Education Mathematics Curriculum Standard (Ministry of Education of the People's Republic of China, 2022)” that factorization can be carried out by using the common factor method and the formula method (directly using the formula not more than twice) (the index is a positive integer). This kind of deformation, in which a sum form is transformed into a product form in the real number range, will help cultivate students' observation ability, operation ability, creativity, etc. Because of its strong technical and educational value (Xu, 2018), it plays an important role in the junior high school mathematics system. Since cognitive psychologists have not yet developed a suitable cognitive model for achievement tests, researchers can construct cognitive models based on their own diagnostic goals. Some scholars have divided the cognitive attributes involved in factorization into five items: A1: the concept of factorization; A2: the concept of the common factor; A3: the extracting the common factor method; A4: the method of the square difference formula; and A5: the method of the perfect square formula. A1 and A2 are basic concepts belonging to the first layer. A3, A4, and A5 are specific methods for the second layer. A1 is the basis of A3, A4, and A5. One-way arrows exist from A1 to A3, A4, and A5. A2 is the basis of A3. There is a one-way arrow from A2 to A3. Except for the above arrows, no arrow connects the residual attributes.

However, there is no unrelated relationship between the method of extracting the common factor, the method of the square difference formula, and the method of the perfect square formula. This point is described in detail in a study by Xu (2017), in which some algebraic polynomials are regarded as research objects and the multiplication formulas using inverse polynomials are regarded as research tools. The purpose is to decompose a polynomial into the product of several factors. In this sense, the method of extracting the common factor, the method of the square difference formula, and the method of the complete square formula are all research tools; therefore, it is reasonable for some scholars to regard them as being on the same level. However, educational mathematics uses the most basic “grouping-extracting common factor” as a research tool. The method of extracting a common factor refers to extracting the common factor that can be directly observed in the polynomial without grouping. Therefore, it is the basis of the method of “grouping-extracting common factors.” The square difference formula method and the complete square formula method can be derived using the method of “grouping-extracting the common factor” by adding polynomials and extracting the common factor by grouping. Therefore, the method of extracting the common factor is the basis of the method of square difference formula and the method of perfect square formula, and these three methods can be merged under the method of grouping-extracting common factors. The premise of the method of grouping-extracting common factors is “grouping,” which reflects students' logical reasoning, visual imagination, mathematical operation ability, and other abilities. To describe the cognitive model in more detail, the method of grouping-extracting common factors is expressed by grasping the different levels of ability of the method of extracting the common factor. Therefore, the knowledge attributes of factorization have five items: A1: the concept of factorization; A2: the concept of the common factor; A3: the method of extracting the common factor; A4: the method of the square variance formula, and A5: the method of the perfect square formula.

## Establishment of the factorization cognitive model

The concepts of factorization (A1) and the common factor (A2) should be on the level of knowledge understanding (B1) after analyzing the capability dimension and knowledge dimension of factorization. The extracting common factor method (A3) has different levels of cognitive operation and different requirements for students. It can be divided into three levels: knowledge understanding (B1), knowledge transfer (B2), and knowledge innovation (B3). Among them, A3B1 refers to the knowledge understanding of extracting the common factor method, such as extracting the common factor that can be directly observed in the polynomial. A3B2 refers to the knowledge transfer of the extracting common factor method. The so-called “knowledge migration” refers to the ability to carry out cross-context migration of basic knowledge and basic skills and integrate multiple types of knowledge. For example, to solve the decomposition of some quadratic trinomials, the method is called cross-multiplication in the textbook. In fact, cross multiplication is also a special case of the method of “grouping-extracting common factors.” However, it would be more difficult than the “formulation method” to use. A3B3 refers to the knowledge innovation method, and the “innovation” is relative to the learner. For a learner, if the new knowledge and new methods have transcended the textbook's content, the new knowledge should be obtained through self-discovery. The method of the square variance formula (A4) and the method of the perfect square formula (A5) are both applications of the formula, which can be regarded as the special case of the method of “grouping-extracting common factor.” Thus, they should belong to the level of knowledge understanding (B1). The level of competence contained in each knowledge content is given in Table 1 below (1 means knowledge-implied ability and 0 means no implication).

**Table 1:** The level of competence contained in each knowledge content of factorization.

**Knowledge attribute and ability level**	**Knowledge understanding B1**	**knowledge transfer B2**	**Knowledge innovation B3**
Concept of factorization A1	1	0	0
Concept of common factor A2	1	0	0
Method of extracting the common factor A3	1	1	1
Method of the square variance formula A4	1	0	0
Method of the perfect square formula A5	1	0	0

Through discussions with educational experts, it has been determined that the cognitive factorization model involves seven attributes: A1B1: knowledge understanding of the factorization concept; A2B1: knowledge understanding of the concept of the common factor; A3B1: knowledge understanding of the method of extracting the common factor; A3B2: knowledge transfer of the method of extracting the common actor; A3B3: knowledge innovation of the method of extracting the common factor; A4B1: knowledge understanding of the method of the square variance formula; and A5B1: knowledge understanding of the method of the perfect square formula (see Table 2 for specific performance).

**Table 2:** Factorization cognitive model attribute.

**Code**	**Full name**	**Description**
A1B1	Knowledge-understanding of the factorization concept	Understand and master the basic concepts of factorization
A2B1	Knowledge understanding of the concept of the common factor	Understand and grasp the concept of the common factor, be able to determine the common factor of the polynomial
A3B1	Knowledge understanding of the method of extracting the common factor	Proficiently and accurately decomposing polynomials whose common factor that can be observed directly
A3B2	Knowledge transfer of the method of extracting the common factor	Extracting the common factor by grouping the polynomials and using A3, A4, and A5 comprehensively
A3B3	Knowledge innovation of the method of extracting the common factor	Decomposing polynomials through adding terms and extracting the common factor of the terms by grouping.
A4B1	Knowledge understanding of the method of the square variance formula	Proficiently and accurately decomposing polynomials by the method of formula for the difference of square
A5B1	Knowledge understanding of the method of the perfect square formula	Decomposing proficiently and accurately polynomials by the method of formula for the perfect square

**Figure 1:**
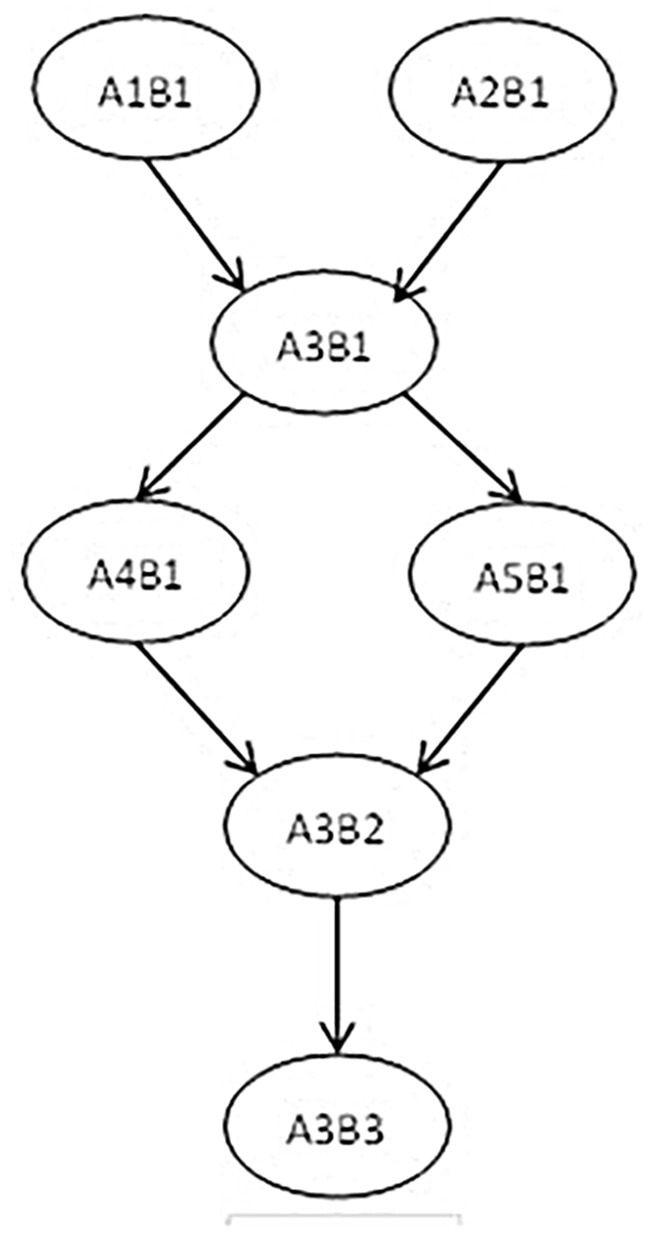
Factorization of hierarchical relationships between cognitive attributes.

Leighton et al. (1999) believed that cognitive attributes are not independent operations but subordinate to an interrelated network. There may be a certain logical order or hierarchical relationship between cognitive attributes. The cognitive model is a hierarchical relationship diagram used to represent related tasks. We constructed a hierarchy of seven attributes related to the cognitive factorization model, as shown in Figure 1. The following hierarchical relationship diagram reflects the order of psychological processing and cognitive development of individuals in mastering factorization knowledge to a certain extent.

## Empirical research

### Research I: rationality of factorization cognitive model

#### Research purposes

To test the rationality of the cognitive model through verbal reports and the regression of the item's difficulty on the cognitive attributes.

#### Research methods

Research ideas (Cai and Tu, 2015): First, according to the cognitive factorization model, we would have the adjacency matrix (a matrix reflecting the direct relationship between attributes) and the reachability matrix R (a matrix reflecting the direct relationship, indirect relationship, and self-relations between the attributes), and the ideal mastering pattern and typical project evaluation pattern would be obtained after it. Then, after designing the test matrix Q (Tatsuoka, 1983), which is a structured representation of the relationship between items and potential attributes (Wang et al., 2018) (generally, the test Q matrix should include the typical project evaluation patterns), based on the typical project evaluation pattern, the test questions would be compiled and organized accordingly. Third, eighth graders from two junior high school classes in Wuhan were selected for testing. Eight students from each subject were selected to report on the 15th, 19th, and 20th questions in the test. Then, after collecting the answer sheet data, sorting it by 0-1, calculating the difficulty of each item, and using R statistical software to build a program to establish a regression analysis of the project's difficulty on cognitive attributes to verify the rationality of the cognitive model (Because of the page limit, only the test matrix Q is shown here, omitting the adjacency matrix A, the reachability matrix R, the ideal mastering mode, and the typical project evaluation mode)


$$\begin{array}{l} Q\\ \text{​​​​​​}=\;\left|\begin{array}{lllllllllllllllllllll} \; & 1 & 2 & 3 & 4 & 5 & 6 & 7 & 8 & 9 & 10 & 11 & 12 & \text{​}13 & \text{​}14 & \text{​}15 & \text{​}16 & \text{​}17 & \text{​}18 & \text{​}19 & \text{​}20\\ A1B1 & 1 & 0 & 1 & 1 & 1 & 1 & 1 & 1 & 1 & 1 & 1 & 1 & 1 & 1 & 1 & 1 & 1 & 1 & 1 & 1\\ A2B1 & 0 & 1 & 1 & 1 & 1 & 1 & 1 & 1 & 1 & 1 & 1 & 1 & 1 & 1 & 1 & 1 & 1 & 1 & 1 & 1\\ A3B1 & 0 & 0 & 0 & 1 & 1 & 1 & 1 & 1 & 1 & 1 & 1 & 1 & 1 & 1 & 1 & 1 & 1 & 1 & 1 & 1\\ A4B1 & 0 & 0 & 0 & 0 & 1 & 0 & 1 & 0 & 1 & 0 & 1 & 1 & 1 & 1 & 1 & 1 & 1 & 1 & 1 & 1\\ A5B1 & 0 & 0 & 0 & 0 & 0 & 1 & 0 & 1 & 1 & 1 & 1 & 1 & 1 & 1 & 1 & 1 & 1 & 1 & 1 & 1\\ A3B2 & 0 & 0 & 0 & 0 & 0 & 0 & 0 & 0 & 0 & 0 & 0 & 0 & 0 & 1 & 1 & 1 & 1 & 1 & 1 & 1\\ A3B3 & 0 & 0 & 0 & 0 & 0 & 0 & 0 & 0 & 0 & 0 & 0 & 0 & 0 & 0 & 0 & 0 & 0 & 0 & 1 & 1 \end{array}\right| \end{array}$$

#### Sample method and subjects

The random sampling method was used to extract samples from eighth-grade students taught by the author. A total of 72 students were randomized from two classes in the eighth grade of a junior high school in Wuhan. Eight students from it were selected for verbal reports. The eighth-grade students had mastered basic algebraic knowledge, such as rational numbers and real numbers, and also learned the first-order linear equation. They also gained skills in dealing with inequality and binary linear equations.

#### Measurement tools

We prepared factorization test questions. There were 20 questions (eight multiple-choice questions, four fill-in-the-blank questions, and eight answer questions), of which the 15th and 19th questions were used as “anchor questions.” Questions 1–13 were designed to examine the level of knowledge understanding B1, questions 14–18 were designed to examine the level of knowledge transfer B2, and questions 19–20 were designed to examine the level of knowledge innovation B3. The examinations of attributes A1B1, A2B1, A3B1, A4B1, A5B1, A3B2, and A3B3 involved 19 questions, 19 questions, 17 questions, 13 questions, 14 questions, 7 questions, and 2 questions, and the order of the questions was arranged from easy to difficult. Examples of self-edited questions are as follows:

Question 19. Read the following materials and answer the questions:

When Xiao Ming observed the equation (*x*+*p*)(*x*+*q*) = *x*^2^+(*p*+*q*)*x*+*pq*, it was found that, when *p* is opposite to *q*, the square variance formula can be obtained. When *p* is the same with *q*, the perfect square formula can be obtained. The process of Xiao Ming's decomposition of *x*^2^−*y*^2^ is given: *x*^2^−*y*^2^ = *x*^2^−*xy*+*xy*−*y*^2^ = *x*(*x*−*y*)+*y*(*x*−*y*) = (*x*−*y*)(*x*+*y*). Can you refer to this idea and decompose factor:*x*^3^−*y*^3^.

#### Research result I

The test of verbal reports (take the 19th question as an example, two students' reports were selected for display): Student 1: “According to the materials, I write down *x*^3^−*y*^3^, try to add a middle item and subtract the same item, then divide them into two groups and merge the similar items, which have the common factor.” The answering process for Student 1 is as follows:


$$\begin{array}{cc} 解:原式=x^{3}\;-\;y^{3}\;\\ = & \;x^{3}\;-\;x^{2}y\;+\;x^{2}y\;-\;y^{3}\;\\ = & \;x^{2}\left(x\;-\;y\right)\;+\;y\left(x^{2}\;-\;y^{2}\right)\;\\ = & \;x^{2}\left(x\;-\;y\right)\;+\;y\left(x\;+\;y\right)\left(x\;-\;y\right)\;\\ = & \;\left(x^{2}\;+\;xy\;+\;y^{2}\right)\left(x\;-\;y\right) \end{array}$$

Student 2: “According to the materials, adding and subtract all items, including *x, y* with a lower degree, and then extract the common factor.” The answering process of student 2 is as follows:


$$\begin{array}{cc} 解:原式=\;x^{3}\;-\;y^{3}\;\\ = & \;x^{3}\;+\;x^{2}y\;+\;x^{2}y\;-\;y^{3}\;\\ = & \;x\left(x^{2}\;+\;xy\;+\;y^{2}\right)\;-\;y\left(x^{2}\;+\;xy\;+\;y^{2}\right)\\ = & \;\left(x^{2}\;+\;xy\;+\;y^{2}\right)\left(x\;-\;y\right) \end{array}$$

It can be clearly observed from the students' answering process that they need to try to add and subtract some items when solving the 19th question, grouping the polynomials and extracting the common factors from the polynomials after the grouping. It also indicates that the student's thinking process is consistent with the basic idea of educational mathematics in dealing with the factorization problem, but the hierarchical relationship between attributes cannot be accurately verified. In this process, the hierarchical relationship of attributes between A3B1, A3B2, and A3B3 is reflected, but the hierarchical relationship of attributes between A1B1, A2B1, A3B1, A4B1, and A5B1 is not reflected. There are two main reasons for this: The first is the limitations of verbal reports.

Since knowledge can be divided into procedural and declarative knowledge, such as A1 and B1 being declarative knowledge, it obviously cannot be revealed in the student's answering process to this question. However, it does not mean that the student's understanding of the concept of factorization and the concept of the common factor is not examined. If students do not understand these basic concepts, they may not understand the meaning of the question, and they would not answer correctly. Therefore, sometimes, it is difficult for some declarative knowledge to be tested by verbal reports. Second, the cognitive structure of students differs from the established cognitive model. From the perspective of educational mathematics, the cognitive factorization model is established through a deeper understanding of the teaching content. Therefore, it is more suitable for students with higher ability levels. The cognitive structure of factorization in students is largely influenced by teachers' mathematical pedagogical content knowledge (MPCK), but not all mathematics teachers' cognitive structures are consistent with those advocated for educational mathematics.

**Figure 2:**
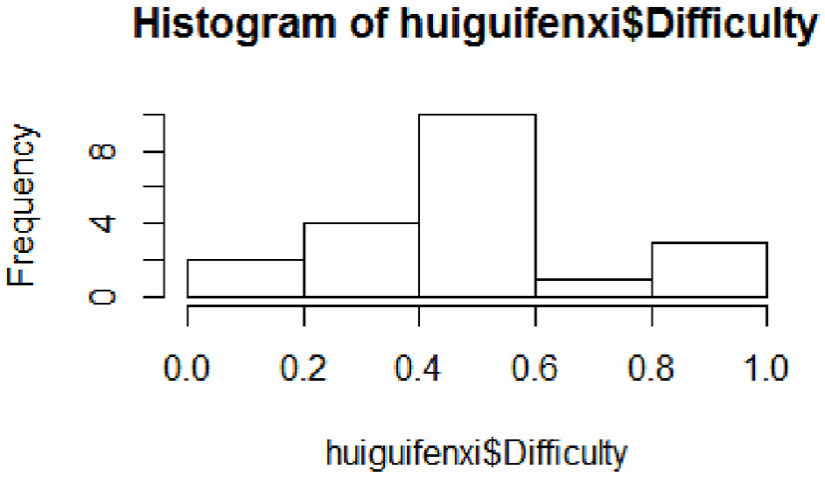
Project difficulty frequency distribution histogram.

Regression analysis: By establishing the regression of the project difficulty to the cognitive attributes, the degree of interpretation of the cognitive attributes was examined to test the completeness of the cognitive attributes. The seven attributes A1B1, A2B1, A3B1, A4B1, A5B1, A3B2, and A3B3 were used as independent variables, and the project difficulty was used as the dependent variable to establish the regression equation. The specific process: First, plot the frequency distribution histogram to detect the distribution of the difficulty of the dependent variable, as shown in Figure 2.

**Figure 3:**
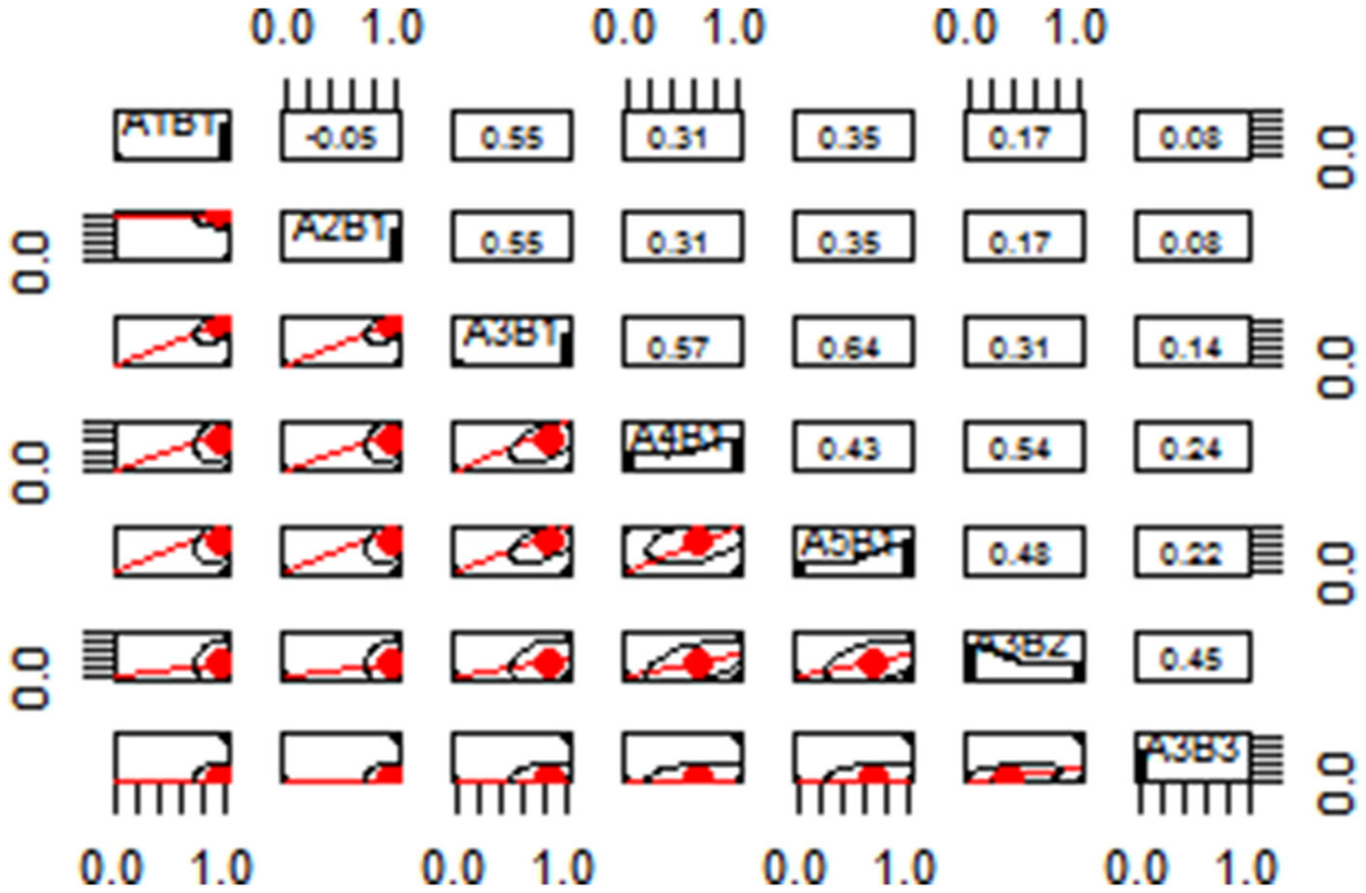
Scatter plot matrix.

As is shown in Figure 2, the difficulty of the items varies between (0, 1), the number of items between (0, 0.2) and (0.8, 1) is less, and the difficulty of most items is concentrated between (0.4, 0.6), indicating that the project is more reasonable. The second limitation is calculating the correlation coefficient between each variable and displaying it in the scatter plot matrix, as shown in Figure 3. The scatter plot matrix is represented by the correlation coefficient matrix and the histogram. The correlation coefficient matrix is above the diagonal line. The histogram was used to plot the numerical distribution of each variable on the diagonal. The scatter plot below the diagonal visualizes the correlation between the two variables.

The third limitation is to establish multiple linear regression equations. The results are as follows:


$$\begin{array}{c} y=0.4583x_{1}+0.4167x_{2}-0.1328x_{3}+0.2131x_{4}+0.0011x_{5}\\ -0.1147x_{6}+0.4778x_{7}-0.3889 \end{array}$$

According to the above formula, we could observe the relationship between each attribute and the dependent variable. The variable *x*_7_ indicates that the attribute A3B3 has higher requirements for the student's ability level. The items containing this attribute are difficult, so the variable has a greater impact on the difficulty. Moreover, the last step is the performance evaluation of the model. The multivariate *R*^2^ value of the model is 0.8258, and the adjusted *R*^2^ value is 0.7241, which means that the identified attribute can explain 72.41% of the difficulty of the project. It is generally considered that to reach more than 60% means that the identified cognitive attributes are reliable (Tu et al., 2012).

From the results of the verbal reports and the regression analysis, the attributes in the cognitive factorization model are complete, and the hierarchical relationship between the attributes is partially verified. Thus, the hierarchical relationship between the three different ability levels of extracting the common factor is verified. For some attributes that fail to detect the hierarchical relationship in a verbal report, such as attribute A1B1, attribute A2B1, attribute A3A1, attribute A4B1, and attribute A5B1, by analyzing the correlation between two attributes, there is a highly weak correlation between A1B1 and A2B1 so that the two can be juxtaposed into the same layer. A1B1 and A2B1 are highly positively correlated with A3B1 but moderately correlated with A4B1 and A5B1, and A3B1 is highly correlated with A4B1 and A5B1. Therefore, A3B1 can be regarded as the second layer, and A4B1 and A5B1 are considered as the third layer, since the correlation coefficients between A3B2 and A4B1 or A5B1 are greater than the correlation coefficients between A3B3 and A4B1 or A5B1. A3B2 and A3B3 can be regarded as the fourth and fifth layers, respectively. From this, the cognitive model can be considered to be reasonable.

### Research II: cognitive diagnosis using the cognitive factorization model

#### Research purposes

To diagnose the mastery of factorization knowledge and the level of mathematical operation ability of the eighth graders according to the cognitive factorization model.

#### Research method

Research ideas: Students' response patterns can be divided into ideal (IRT) and observation response patterns. In theory, the researcher calculates the reachability matrix R after obtaining the adjacency matrix A according to the cognitive factorization model and then uses the augment algorithm (Yang et al., 2008) to obtain the ideal test pattern, the ideal master pattern (IMP), and the ideal response pattern (no guessing or error-free response pattern). The test Q matrix is compiled according to the ideal test pattern; that is, when the cognitive model is determined, the ideal reaction pattern can be obtained by Boolean operation, as shown in Table 3.

**Table 3:** The ideal patterns (seven attributes).

**Subjects**	**Ideal master pattern (IMP)**	**Ideal test pattern**	**Ideal response patterns (IRT)**
1	0000000		000000000
2	1000000	1000000	100000000
3	0100000	0100000	010000000
4	1110000	1110000	111000010
5	1111000	1111000	111100010
6	1110100	1110100	111110010
7	1111110	1111110	111111011
8	1111111	1111111	111111111
9	1100000	1100000	110000010
10	1111100	1111100	111110011

The observation response pattern refers to the student's actual response through the observed response pattern that can be obtained directly, expressing students' knowledge state that cannot be observed directly (Tatsuoka, 1995). Owing to the students' guess and slip on the test, the students' observation response patterns were not necessarily ideal, and there were many non-ideal response patterns (Cao, 2009). Therefore, in the cognitive diagnosis, it is also necessary to select an appropriate diagnostic model and estimate the attribute master pattern of the subject according to the diagnostic model, the observation reaction pattern of the subject, and the test Q matrix.

The specific process was, first, data collation. The answer data of 72 subjects were collated to form a 0–1 answering matrix of 72 rows and 20 columns (The correct answer is marked 1, and error or not answered is marked 0). Second, model selection and parameter estimation. The DINA model (Junker and Sijtsma, 2001) (the deterministic inputs, noise “and” gate model) was selected as a diagnostic model for its simplicity and less affected by the number of cognitive attributes (Cai et al., 2013). Many studies have shown that it has high diagnostic accuracy (Cheng, 2008; Rupp and Templin, 2008). We uploaded the Q matrix and the student answer matrix to the cognitive diagnosis analysis platform (http://www.psychometrics-studio.cn/). We chose the DINA model in the “Parameter Estimation” column to estimate parameters. The students attribute mastery probability, and the mastering attribute pattern will follow. Third, we performed a model-fitting test. We then investigated the fitness of subjects and projects. The Lz index was used for the index of fitness of subjects and the Maximum Statistics indicator was used for the project fitting index. Fourth, we conducted a testing quality analysis before calculating the clone Bach coefficient to verify the reliability of the test item. Finally, we analyzed the students' knowledge states and the level of mathematical operating ability according to their attribute master pattern.

#### Research result II

Test reliability, validity, difficulty, and discrimination: The Cronbach's alpha of this test was 0.84, indicating a high degree of consistency between projects, and items could be used to test students' mathematical operating ability. Moreover, once the cognitive model and the diagnostic test are consistent, the test validity would be more fully guaranteed (Ding et al., 2012). We employed construct validity to test validity and the statistical software SPSS to analyze student testing data using exploratory factor analysis (72 subjects and 20 test questions). We also used principal component analysis, determined factor extraction if the eigenvalue was >1, and conducted factor analysis using the orthogonal rotation maximum variance method. The results showed that the number of KMO sampling appropriateness was 0.741, indicating that it was suitable for factor analysis; the Barthes case sphericity value was 578.918, and the significance was 0.000, which was < 0.05, that is, the correlation matrix was not a unit matrix so factor analysis could be carried out. Seven factors were extracted in factor analysis, and the variance contribution rate was 70.946%. Therefore, these data were suitable for factor analysis. Comparing these seven factors and their corresponding observed variables with the test Q matrix, the structure was basically the same; thus, the 7 factors are A1B1, A2B1, A3B1, A4B1, A5B1, A3B2, and A3B3, and the Q matrix was equitable. The difficulty associated with the test is mostly concentrated between 0.4 and 0.6 (Figures 1–**4**). Items with discrimination of less than 0.2 need to be eliminated, and those with discrimination greater than or equal to 0.4 are the best; the rest needs to be improved. The individual test items belong to the basic questions, and the students have a good grasp, so the item discrimination is low, but most of the item discrimination is ≥0.4, and the overall test preparation is reasonable.

Model fitting: The subject fitting index follows the traditional Lz statistic of the item response theory (IRT), and its value is < -2, which means bad fitting. The experimental results showed that four of the 72 subjects' Lz values are < -2. However, 94% of the subjects are still fitted. The project fitting index is based on the Maximum Statistics indicator (see Table 4 below): it is generally considered that “adj.*p*-value.max[z.r] > 0.05” means fitting well.

**Table 4:** Items fitting index.

**Item**	**1**	**2**	**3**	**4**	**5**	**6**	**7**	**8**	**9**	**10**
adj.pvalue.max[z.r]	0.133	0.342	1	0.684	0.247	0.608	0.247	0.684	0361	1
Item	11	12	13	14	15	16	17	18	19	20
adj.pvalue.max[z.r]	0.076	1	0.076	0.855	0.646	0.133	1	0.133	1	0.361

The results of the students' cognitive attributes: Estimating the probability of mastery of each attribute in the student's knowledge state (DINA model) is shown in Table 5.

**Table 5:** Mastery probability of each attribute in the student's knowledge state.

**Number of people**	**A1B1**	**A2B1**	**A3B1**	**A4B1**	**A5B1**	**A3B2**	**A3B3**
72	0.79	0.87	0.82	0.81	0.66	0.71	0.28

As can be observed from Tables 3, 4, the students have a poor grasp of the knowledge attribute A5. The grasp of the first level of attribute A3 is great, the second level of attribute A3 follows, and the third level is badly mastered. The average value of the mastery probability for the attributes A1B1, A2B1, A3B1, A4B1, and A5B1 is 0.79; therefore, the probability of grasping each attribute of the student's mathematical operating ability level can be obtained, as is shown in Table 6.

**Table 6:** Mastery probability of each attribute of mathematical operating ability level.

**Number of people**	**B1**	**B2**	**B3**
72	0.79	0.71	0.28

Approximately 79% of the 72 students reached the level of knowledge understanding, 71% achieved the level of knowledge transfer, and only 28% attained the level of knowledge innovation.

Diagnostic results of students' cognitive status: According to the diagnosis results, some students' knowledge status belonged to the ideal mastery pattern, but some students' knowledge status did not exhibit the ideal master pattern (non-ideal mastery state). The diagnosis types in Table 7 included students' ideal mastery pattern and students' non-ideal master pattern (represented by “^*^”).

**Table 7:** Diagnostic results of students' cognitive status.

**Type of diagnosis**	**Knowledge dimension**	**Ability dimension**	**Number of people**
0000000	0	0	0
1000000	A1	B1	0
0100000	A2	B1	0
1100000	A1, A2	B1	0
1110000	A1, A2, A3	B1	2
1110100	A1, A2, A3, A5	B1	3
1111000	A1, A2, A3, A4	B1	1
1111100	A1, A2, A3, A4, A5	B1	1
1111110	A1, A2, A3, A4, A5	B1, B2	31
1111111	A1, A2, A3, A4, A5	B1, B2, B3	5
0010100^*^	A3, A5	B1	1
0011000^*^	A3, A4	B1	1
0011100^*^	A3, A4, A5	B1	1
0010010^*^	A3	B1, B2	3
0010111^*^	A3, A5	B1, B2, B3	1
0011011^*^	A3, A4	B1, B2, B3	1
0010101^*^	A3, A5	B1, B3	2
0011101^*^	A3, A4, A5	B1, B3	1
0011110^*^	A3, A4, A5	B1, B2	1
0001011^*^	A4	B1, B2, B3	1
0001001^*^	A4	B1, B3	1
0001101^*^	A4, A5	B1, B3	1
0001111^*^	A4, A5	B1, B2, B3	1
0000101^*^	A5	B1, B3	1
0000100^*^	A5	B1	1
1101001^*^	A1, A2, A4	B1, B3	1
1101000^*^	A1, A2, A4	B1	1
1100001^*^	A1, A2	B1, B3	1
1101011^*^	A1, A2, A4	B1, B2, B3	2
1110001^*^	A1, A2, A3	B1, B3	1
1111011^*^	A1, A2, A3, A4	B1, B2, B3	1
1111010^*^	A1, A2, A3, A4	B1, B2	1
1111001^*^	A1, A2, A3, A4	B1, B3	1
1111101^*^	A1, A2, A3, A4, A5	B1, B3	2

The ^*^ symbol indicates students' non-ideal master pattern.

The cognitive status of 43 of the 72 students can be classified as an ideal master pattern, and the cognitive status of 29 students is a non-ideal master pattern. The students in each state were diagnosed based on their knowledge and ability dimensions. For example, students with a cognitive status of 1110100 have attributes A1B1, A2B1, A3B1, and A5B1, so they have attributes A1, A2, A3, and A5 in the knowledge dimension, and the operating ability reaches level B1; students with a cognitive status of 0001001^*^ have attributes A4B1 and A3B3. The students mastered A3B3 in the model but did not master A3B1. Thus, they did not have attribute A3. Although B2 and B3 are theoretically related to the knowledge attribute A3, in fact, B2 and B3, respectively, contain the migration and innovation of the five knowledge attributes; therefore, the students have reached the B3 level probably because A4 is better. Therefore, the student is likely to reach the B3 level because knowledge attribute A4 is well mastered. Therefore, attribute A4 is mastered in the knowledge dimension, and the ability level reaches B1 and B3. Generally speaking, students' ability levels should range from low to advanced. Only when they master the low level is it possible to master the advanced level, but the empirical data show that there are 12 students with the ability level mastering pattern “B1, B3” among the 72 students, and there is an “overstepping” phenomenon. A total of 18 students have mastered knowledge attributes A3, A4, and A5 but not A1 and A2. There are four possible reasons for this phenomenon. First, the loss of some information by means of a 0–1 score results in an inaccurate judgment of the attributes of students. Second, even if the judgment is correct, the development of a student's ability level may not strictly follow the process from knowledge understanding to knowledge transfer and knowledge innovation. Moreover, it may appear that there is a phenomenon of “transition” from knowledge understanding to knowledge innovation.

Third: Since attributes A1 and A2 are declarative knowledge, attributes A3, A4, and A5 are procedural knowledge. Students undergo numerous procedural exercises in their studies, and they have a better mastery of procedural knowledge. Fourth, there is research that indicates that the DINA model is susceptible to the test and sample size (Tu et al., 2012). The small number of samples in this experiment is also likely to reduce the accuracy of the DINA model.

## Limitation and prospects

The diagnostic status of 29 of 72 subjects was not in the ideal mastery status, which reflected that students had a better mastery of the factorization formula but worse mastery of the concept of the factorization formula. A “bypass” phenomenon affects a small part of students' operational ability levels. The main reasons are as follows: First, the use of 0–1 scoring enabled the loss of part of the information, which may have contributed to the student's inability to understand the judgment. Some studies showed that sample size and subjects easily affect the DINA model (Qin et al., 2014). Because this test had fewer samples, the DINA model's classification accuracy rate would be lower (Zhan et al., 2016); second, because attributes A1 and A2 were declarative knowledge, and attributes A3, A4, and A5 were procedural knowledge, students had many declarative exercises in their study process to improve their declarative knowledge mastery. Students often made mistakes due to their incomprehension of the meaning of factorization; the third is the test Q matrix's impact on cognitive diagnosis results. Setting the correct Q matrix is the key factor for obtaining accurate parameter estimation results (Nájera et al., 2020), but these test attributes in the test project theory do not strictly follow the cognitive attributes' hierarchical structure in the cognitive model. The cognitive model reflects subjects' logic sequence, which they must obey in the process of knowledge mastery, but the project test mode does not necessarily follow this logic sequence. For example, question 19 should have examined all attributes in terms of theory, but it primarily tested A3B3 in actual measurement, which is consistent with the result of factor analysis; fourth, if the judgment is correct, the development of student ability level in this process may not strictly accord to knowledge understanding, knowledge transfer, and knowledge innovation. A “transition” may occur from knowledge understanding to knowledge innovation. This point needs more elaborate measurements to be tested; fifth, as students' cognitive results are related to their order structure of knowledge learned and teachers' knowledge structure of teaching lectures, a study shows that teachers' subject knowledge is significantly positively correlated with students' academic performance, which is an important implicit factor affecting students' academic performance (Liu et al., 2016). A cognitive model from the perspective of educational mathematics was established to provide a deeper understanding of teaching content. It is more suitable for students with greater abilities. Moreover, students' cognitive structures for factorization are greatly influenced by teachers' mathematics teaching knowledge, and not all math teachers' cognitive structures are consistent with the structures that educational mathematics advocates. However, when the majority of pupils have the same issue, the unjustified school curriculum may also be to blame (Tu et al., 2012). Additionally, this study is localized in that the testing materials examined students' use of the add-on method to perform factorization, including the “cross multiplication” method. However, the “cross multiplication” method occurs in the reading materials of eighth-grade mathematics textbooks, which is not compulsory content and will not occur in eighth-grade achievement tests. In addition, some students have mastered this method through after-class study, leading to deviations in the results. If the test material is closer to the student's academic test, then the results may be better. Testing content is relatively simple, and factorization is mainly examined so that students' mathematical operation abilities cannot be fully reflected. Knowledge and ability dimensions have been considered when designing the Q matrix, but the cognitive model still uses the DINA model with 0–1 scoring. The assessment of students' ability levels depends on the knowledge mastery results, so the given measurement results of students' ability levels are relatively sweeping. Some students may not even understand the required knowledge, but the model fails to reflect it. The idea of a Q matrix of multilevel attributes is proposed by De la Torre et al. (2010) and Chen and de la Torre (2013), which can carefully examine the project measurement attribute and its level. In other words, using the Q matrix of multilevel attributes can not only display the knowledge attribute of factorization but also the needed ability level of each knowledge attribute. Moreover, each knowledge attribute corresponds to more than one capability level to achieve a more refined measurement of capability level. The generation of the idea of the Q matrix of a multilevel attribute has provided the theoretical basis for the establishment of a multilevel cognitive model. Studies (Cai et al., 2010; Li, 2016) have shown that the stability and parameter estimation accuracy of the attribute multilevel cognitive model is good, and the model accuracy is great. Moreover, this study has provided a practical reference for the establishment of a multilevel cognitive model, which is favored in developing a multilevel attribute cognitive model suitable for students' academic achievement tests.

## Conclusion

ResearchIshows that the construction of the cognitive factorization model is reasonable. ResearchIIshows that approximately 79% of students achieved the level of knowledge understanding, 71% of students reached the level of knowledge transfer, and only 28% of students reached the level of knowledge innovation. Although both studies have shortcomings, the research results were not exactly the same as the predictions but were consistent with the teaching practice. It could be found that, in the actual teaching, if too much attention is paid to the “problem-solving practice” of procedural knowledge but not to the understanding of declarative knowledge, it will be detrimental to the cultivation of students' knowledge transfer ability. This is also the reason why attribute B2 is not listed separately when judging the level of students' ability in the second study. Knowledge transfer and knowledge innovation may not necessarily be strict subordinate relationships, but it is certain that, if students do not reach the level of knowledge understanding, it is difficult to transfer knowledge. Although some researchers pointed out that the concept of factorization should be diluted (Yu J., 2018), this does not mean that the conceptual teaching of factorization can be skipped directly. Huaishui Zhou, Shoulei Chen, and other researchers studied the errors in the student factorization test and found that some students were mistaken because they did not understand the meaning of factorization. An accurate understanding of concepts is the basis for developing students' ability to transfer knowledge.

Although most students can achieve knowledge understanding and knowledge transfer, only a small number of students can reach the level of knowledge innovation. According to research by Dyers et al. (2011), “Two-thirds of human creativity comes from education, and one-third comes from inheritance. On the contrary, in intelligence, one-third is from education, and two-thirds is from heredity. Therefore, we may not be able to do a lot to improve one's intelligence, but we can cultivate their creativity”. Teachers' mathematical pedagogical content knowledge greatly influences students' cognitive structure. Thus, how can teachers assist students in moving from the level of knowledge transfer to the level of knowledge innovation?

Educational mathematics points the way. Knowledge innovation refers to the ability to analyze and judge things in a mathematical way, as well as to know the world with mathematical thinking and vision. Along the technical route of educational mathematics, “researching problems-research objects-research tools,” students' thinking styles and thinking habits can be better cultivated. Problem-solving teaching is a part of mathematics teaching, and mathematics thinking methods can be well infiltrated into students through problem-solving teaching, but teachers should not be limited to existing mathematical thinking methods. Otherwise, it will limit the development of students' creativity. If we regard these mathematical thinking methods and mathematical knowledge as tools and means to solve research problems, then we can encourage students to explore new methods or use new methods to solve new problems creatively. Educational mathematics pays more attention to the students' thinking mode and allows students to generate mathematical thinking methods by thinking about the process of solving specific problems (Franke et al., 2001). It is this thinking that guides students to acquire mathematics naturally. In this way, students can comprehend not only mathematical thinking methods and creatively propose ways to solve problems but also learn a way and habit of thinking about problems. If knowledge is the origin of the core literacy generation (Fu, 1994, pp. 80–85), education mathematics as a mathematical teacher knowledge theory system is the compass to develop students' core literacy.

## Data availability statement

The original contributions presented in the study are included in the article/supplementary material, further inquiries can be directed to the corresponding author.

## Author contributions

CJ: writing—original draft, conceptualization, formal analysis, and visualization. XZ: writing—review and editing, resources, and supervision. LJ: writing—review and editing. LN: formal analysis. LB: methodology. All authors contributed to the article and approved the submitted version.
